# The Use of *In Silico* Tools for the Toxicity Prediction of Potential Inhibitors of SARS-CoV-2

**DOI:** 10.1177/02611929211008196

**Published:** 2021-04-12

**Authors:** Varsha Bhat, Jhinuk Chatterjee

**Affiliations:** Department of Biotechnology, PES University, Bangalore, India

**Keywords:** computational toxicology, Covid-19, *in silico* toxicology, SARS-CoV-2, toxicity prediction

## Abstract

The current strategy for treating the Covid-19 coronavirus disease involves the repurposing of existing drugs or the use of convalescent plasma therapy, as no specific therapeutic intervention has yet received regulatory approval. However, severe adverse effects have been reported for some of these repurposed drugs. Recently, several *in silico* studies have identified compounds that are potential inhibitors of the main protease (3-chymotrypsin-like cysteine protease) and the nucleocapsid protein of SARS-CoV-2. An essential step of drug development is the careful evaluation of toxicity, which has a range of associated financial, temporal and ethical limitations. In this study, a number of *in silico* tools were used to predict the toxicity of 19 experimental compounds. A range of web-based servers and applications were used to predict hepatotoxicity, mutagenicity, acute oral toxicity, carcinogenicity, cardiotoxicity, and other potential adverse effects. The compounds were assessed based on the consensus of results, and were labelled as positive or negative for a particular toxicity endpoint. The compounds were then categorised into three classes, according to their predicted toxicity. Ten compounds (52.6%) were predicted to be non-mutagenic and non-hERG inhibitors, and exhibited zero or low level hepatotoxicity and carcinogenicity. Furthermore, from the consensus of results, all 19 compounds were predicted to be non-mutagenic and negative for acute oral toxicity. Overall, most of the compounds displayed encouraging toxicity profiles. These results can assist further lead optimisation studies and drug development efforts to combat Covid-19.

## Introduction

Since the first case was reported in Wuhan, China, in December 2019,^1^ the Covid-19 coronavirus disease pandemic, caused by the severe acute respiratory syndrome coronavirus-2 (SARS-CoV-2), has led to an unprecedented 120,383,919 cases across the globe as of 17 March 2021.^2^ This is the third coronavirus outbreak in approximately 17 years; the previous outbreaks occurred in China in 2002 (SARS) and Saudi Arabia in 2012 (Middle East Respiratory Syndrome, MERS).^3^ Coronaviruses are enveloped, single-stranded positive-sense RNA viruses belonging to the *Betacoronavirus genus*.^4^ Phylogenetic studies have shown that SARS-CoV-2 is closely related to the bat SARS-like coronavirus, but the intermediate hosts that ultimately led to its transmission to humans have not yet been determined.^4^ However, a recent study suggested that pangolins may be a potential intermediate host.^5^

The infection primarily causes pneumonia-like symptoms, including cough, fever, headache, fatigue and shortness of breath.^6^ Human-to-human transmission occurs by close contact with an infected individual, mainly through the spread of respiratory droplets during coughing or sneezing. Currently, as no specific drugs have received approval,^6^ many existing antiviral and antimalarial drugs are being repurposed for treatment. Antivirals (remdesivir, lopinavir/ritonavir, ribavirin and favipiravir) and antimalarials (chloroquine and hydroxychloroquine) have been recommended for treatment; remdesivir, in particular, has shown promising results.^7^ However, the safety and efficacy profiles of these drugs for treating Covid-19 are still being evaluated in clinical trials, and they have been associated with numerous adverse effects, including gastrointestinal abnormalities (such as diarrhoea and vomiting),^8^ higher risk of liver impairment and cardiac complications.^9^ Another treatment option that is being studied is convalescent plasma therapy, which involves the transfer of convalescent plasma (containing high titres of antibodies against SARS-CoV-2 antigens) from individuals who have recovered from Covid-19 to individuals at high risk (as a prophylactic) or as a means of treatment. Although this therapy has been shown to lead to an improvement in symptoms, the studies performed up to now have involved small sample sizes, and large scale studies are required to establish whether it results in a significant reduction in mortality.^7^

**Figure 1.**
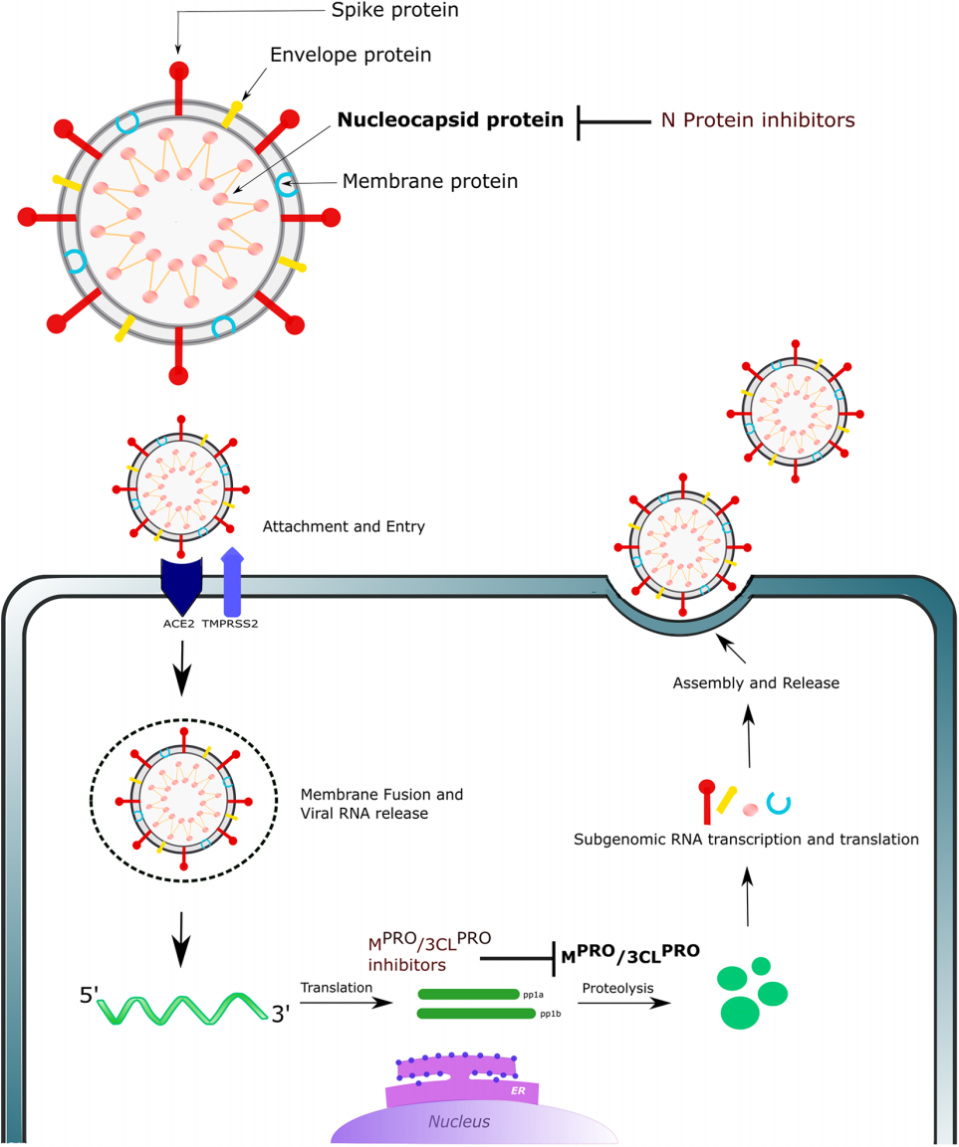
The structure and life cycle of SARS-CoV-2 and the biological targets for inhibition. Note: The colour version of this image is available online.

Several *in silico* studies on the identification of experimental compounds that can specifically target SARS-CoV-2 have been published.^10,11^ The experimental compounds were docked with either the crystal structure or homology-modelled structure of the main protease M^pro^ (also known as 3-chymotrypsin-like cysteine protease (3CL^pro^))^10–12^ or the nucleocapsid protein (N protein)^13^ of the virus (Figure 1). Many of the screened compounds exhibited high docking scores and thus might represent promising treatments. However, toxicity has to be closely monitored during preclinical studies before these compounds can be used. Drug development generally can take around 12 years,^14^ and the preclinical toxicity testing of compounds to evaluate adverse effects and endpoints also has both financial and ethical limitations. Computational toxicity prediction methods can be used to carry out preliminary screening to predict appropriate toxicity endpoints, and thus guide further toxicity tests and compound selection.^15,16^ This approach is particularly helpful in speeding up drug development efforts, which is of paramount importance during health crises such as the current pandemic. A host of tools based on various *in silico* modelling methods, such as structural alerts, read-across and quantitative structure–activity relationships (QSARs), have been developed and are widely used to facilitate the prediction of toxicity.

In this study, a range of *in silico* toxicity prediction tools were used to evaluate the toxicity of some experimental compounds that have been recently reported in the literature as potentially targeting SARS-CoV-2. The results obtained were used to categorise the compounds according to their predicted toxicity profiles.

## Material and methods

A literature search was performed with PubMed to find papers that described the virtual screening of compounds that could potentially target SARS-CoV-2. The keywords used were: ‘Covid-19’, ‘SARS-CoV-2’, ‘docking’, ‘virtual screening’, ‘inhibitors’, ‘treatment’ and ‘therapy’. Based on the search results, 19 top-scoring experimental compounds and their respective targets were identified from four studies published online before 18 April 2020 (Table 1). Of these 19 compounds, 17 were selected from docking studies screening for potential M^pro^/3CL^pro^ inhibitors^10–12^ and two compounds were selected from a study that screened potential N protein inhibitors.^13^ In addition, molecular dynamics simulations and ADME (absorption, distribution, metabolism and excretion) predictions had been completed to assess the dynamic behaviour and drug-likeness of the top hits, respectively, in three of the chosen studies.^11–13^ Among the 19 compounds, 15 belonged to the ZINC15 database,^17^ and four were from PubChem.^18^ The compound pairs ZINC000543523838 and ZINC000543523837, and ZINC000544491494 and ZINC000544491491, are stereoisomers (Table 1).

**Table 1.** The 19 compounds selected for toxicity prediction.

Compound	Reference
ZINC000541677852	10
ZINC000636416501	10
ZINC000543523838^a^	10
ZINC000544491494^b^	10
ZINC000544491491^b^	10
ZINC000541676760	10
ZINC000543523837^a^	10
ZINC000152979101	10
ZINC000152975931	10
ZINC001627499877	10
ZINC001362111980	10
ZINC000003118440	12
ZINC000000146942	12
PubChem CID: 444603	11
PubChem CID: 444743	11
PubChem CID: 444745	11
ZINC001014061081	11
ZINC001014061061	11
PubChem CID: 11610052	13

Stereoisomer pairs are indicated by ^a^ and ^b^, respectively.

For all compounds, the predictions were performed by using web-based servers and applications that had been developed based on distinct approaches, and that had shown considerably high accuracy (≥ 80%) and sensitivity. It was ensured that compounds were within the applicability domain of the respective prediction models of any tools used. Some of the tools displayed a statement directly indicating whether or not a compound was within the applicability domain. In other cases, the applicability domain index was used — with a score of zero indicating that a compound fell outside the applicability domain of that tool. The compounds were primarily assessed based on the following parameters: hepatotoxicity, mutagenicity, acute oral toxicity and carcinogenicity. Hepatotoxicity is one of the leading causes of withdrawal of drugs from the market, and is a key toxicity endpoint that is often assessed during preclinical evaluation.^19^ Mutagenicity and acute oral toxicity testing (as a part of genotoxicity testing) are routinely recommended for pharmaceuticals by regulatory agencies such as the US Food and Drug Administration (FDA).^20,21^ Although FDA guidelines recommend carcinogenicity testing for pharmaceuticals whose expected clinical use is for a minimum of six months,^22^ we performed carcinogenicity predictions to obtain an in-depth toxicity profile for each compound. Qamar et al.^11^ performed toxicity predictions for the compound with PubChem ID 11610052; although their results had predicted that the compound was non-toxic for mutagenicity and carcinogenicity, only one web-based server was utilised in their study. In this study, three different applications/web-based servers were used for toxicity prediction for each of the listed parameters, and a consensus prediction was obtained based on specific parameters, such as probability and the applicability domain of each application, to acquire a more reliable prediction.^15,16^ In addition to the above effects, cardiotoxicity was also assessed. However, as only one web-based server was used, a consensus prediction was not necessary.

### Hepatotoxicity

Hepatotoxicity (referred to as drug-induced liver injury (DILI) in the case of drugs) is a relatively common side effect of numerous medications, in part because the liver receives a relatively high dose of orally dosed drugs from the gastrointestinal tract and acts as the chief site of drug metabolism, potentially resulting in reactive (toxic) metabolites. Alternatively, the first-hand exposure of the liver to toxic drugs that can be metabolised *in situ* to non-toxic derivatives before reaching other organs, also makes the liver a highly vulnerable organ. Adverse hepatic effects can lead to the discontinuation of a drug — in extreme cases, patients might experience acute liver failure and even require liver transplantation.^23^ Three toxicity prediction servers/applications were employed in the current study for the assessment of hepatotoxicity, namely: ProTox-II (version last updated in March 2020);^24^ DL-DILI Prediction Server;^25^ and the VEGA platform (version 1.1.5).^26,27^ ProTox-II and DL-DILI are based on a random forest machine learning algorithm and deep learning algorithm, respectively. On the other hand, the VEGA platform implements a rule-based model based on the determination of structural alerts in the input compound; the alerts are labelled as either toxic or non-toxic.

For the prediction, the canonical SMILES representations of the compounds were provided as input to both ProTox-II and VEGA, while the SDF files of the compounds were provided as the input to the DL-DILI server. The DL-DILI server offers a choice between two models: the DL-Combined model and the DL-Liew model. In this study, the DL-Combined model was selected for the prediction process, due to its higher accuracy, sensitivity and specificity. Both ProTox-II and DL-DILI display whether the compound is positive or negative for hepatotoxicity, as well as the probability associated with the result. VEGA displays the positive/negative result, the applicability domain index, similarity and accuracy indices, and the structural alerts found in the compound. If the compound showed a positive result for hepatotoxicity on two or more servers/applications, it was classified as ‘possibly hepatotoxic’; if not, it was labelled as ‘non-hepatotoxic’. The compounds labelled as ‘possibly hepatotoxic’ were then categorised according to the structural alerts found in the respective compound and the degree of hepatotoxicity associated with similar compounds (for read-across evaluation). Among these, compounds with > 0.8 similarity to previously withdrawn drugs or well-known hepatotoxic compounds/drugs, or with relevant structural alerts (that is, the alerts associated with such hepatotoxic compounds) were additionally labelled as ‘high possibility of hepatotoxicity’. The rest of the compounds were labelled as ‘low possibility of hepatotoxicity’. The hepatotoxicity information about similar compounds was obtained from the LiverTox database.^28^

### Mutagenicity

Mutagenicity testing is primarily based on *in vitro* bacterial and mammalian assays,^29^ such as the Ames assay, where the mutagenicity of a substance is tested with *Salmonella typhimurium*.^30^ ProTox-II, Toxtree^31^ and the CONSENSUS mutagenicity (Ames assay) model, as well as four distinct QSAR models of VEGA (namely, CAESAR, ISS (as implemented in Toxtree), SarPy and KNN), were used for the prediction. Toxtree employs a ‘rule-based’ prediction built on the Benigni/Bossa rulebase for mutagenicity prediction;^32^ on selection of the relevant decision tree, it displays the structural alerts found in each compound. The CONSENSUS model displays a consensus result of the four models — CAESAR, ISS (as implemented in Toxtree), SarPy and KNN.

All three applications were provided with the canonical SMILES representation of the compounds as the input. Toxtree displayed the presence/absence of structural alerts for mutagenicity, while ProTox-II and the VEGA models showed whether a compound is positive or negative for mutagenicity and the associated parameters, such as the corresponding probability on ProTox-II. The consensus approach was also used here for labelling the compounds as ‘possibly mutagenic’ or ‘non-mutagenic’.

### Acute oral toxicity

Acute oral toxicity testing involves the testing of substances to identify any health hazards that might occur upon oral administration, usually after exposure to a single dose or multiple doses within 24 hours. In the past, the rodent acute oral toxicity test was based on LD_50_ determination. However, alternative testing approaches have replaced this traditional LD_50_ testing, due to limitations posed by interspecies variability and various ethical issues.^33,34^

Three web-based servers were used in the current study for the prediction of acute oral toxicity: ProTox-II; DL-AOT Prediction Server;^35^ and admetSAR 2.0.^36^ All three servers employ machine learning-based models and have been trained with large datasets; ProTox-II follows the Globally Harmonised System (GHS) classification scheme, while DL-AOT and admetSAR follow the US Environmental Protection Agency (EPA) classification criteria. The GHS system and the US EPA criteria classify the compounds into five and four categories, respectively.^35,37^

The canonical SMILES representations of the compounds were provided as input for admetSAR and ProTox-II, and the SDF files as input for the DL-AOT server. In addition to the substance’s predicted category, the LD_50_ values are also displayed as part of the results for each compound. While ProTox-II displays the prediction accuracy of the results, DL-AOT and admetSAR show the associated probability. Both DL-AOT and admetSAR use two deep learning models, namely regression and classification, for the prediction of the LD_50_ values and the category, respectively. The classification category and the LD_50_ values were noted for each compound. LD_50_ values vary between different drugs — the LD_50_ of aspirin is 200 mg/kg,^38^ whereas that of acyclovir is greater than 20,000 mg/kg,^39^ when the test organism is the rat. Also, the classification criteria are usually applicable for chemicals other than pharmaceuticals.^37^ Thus, we categorised the compounds as ‘low probability for acute toxicity’ if the LD_50_ values (from the classification category as well) were above 50 mg/kg on two or more servers, which is the upper threshold for high toxicity according to the GHS classification scheme,^37^ and the Loomis and Hayes criteria.^40^

### Carcinogenicity

Carcinogenicity refers to the ability of substances (carcinogens) to induce cancer in humans and experimental animals, through genotoxic or non-genotoxic means.^41^ In this study, we utilised the ProTox-II server, Toxtree’s rule-based model based on the Benigni/Bossa rules,^32^ and VEGA for carcinogenicity predictions. VEGA offers four carcinogenicity models — CAESAR (neural network-based model), ISS (based on the Benigni/Bossa rule-base, as implemented in Toxtree), IRFMN/ANTARES and IRFMN/ISSCAN-CGX (two distinct rule-based models). Hence, CAESAR, IRFMN/ANTARES and IRFMN/ISSCAN-CGX models were selected for prediction on VEGA.

The carcinogenicity predictions were performed in a manner similar to the mutagenicity predictions, with the same input format for all three applications/servers. ProTox-II and the CAESAR model of VEGA predict whether or not a compound is carcinogenic, and the corresponding probability; CAESAR also shows the applicability domain, similarity, and the accuracy indices of the prediction, along with other parameters that were not used in this study. Toxtree shows the structural alerts for genotoxic and non-genotoxic carcinogenicity, if present in the input compound. The other two rule-based models of VEGA display whether a compound is a potential carcinogen or non-carcinogen, the corresponding applicability domain index, structural alerts and other parameters. The consensus of results obtained for the three VEGA models was taken as the result obtained from VEGA for each compound, provided that all three models had values greater than zero for the applicability domain index. In the case of a model’s applicability domain being zero, its result was not considered, and the result was based on the model whose applicability domain index was highest. If there were disparities between the consensus and the result of the VEGA model with the highest applicability domain index, both were noted in order to later compare them with the results from ProTox-II (the probability) and Toxtree (the significance of the structural alerts).

For the overall evaluation, the consensus of all the results was considered; if two or more of the applications/servers predicted a positive result for carcinogenicity, then the compound was labelled as ‘possibly carcinogenic’. On the other hand, if two or more applications/servers showed a negative result for carcinogenicity, the compound was labelled as ‘non-carcinogenic’. Furthermore, if a compound exhibited > 0.8 similarity to previously withdrawn or well-known carcinogenic drugs/compounds, or had relevant structural alerts, it was additionally labelled as ‘high possibility of carcinogenicity’. Information about similar compounds and drugs was obtained from PubChem and a PubMed literature search.

### Other toxicity targets and endpoints

Some of the applications/servers used^24,27,36^ also displayed information on the potential binding of a compound to a few well-known toxicity targets, such as the androgen receptor, aryl hydrocarbon receptor and oestrogen receptor. However, a consensus was not obtained as the results were mostly unreliable. This was because of low applicability domain indices and/or poor prediction accuracy of the models. Hence, the results for binding to the targets were considered only if at least two servers displayed a high probability of binding to a particular toxicity target, and the compound was in the applicability domain.

**Figure 2.**
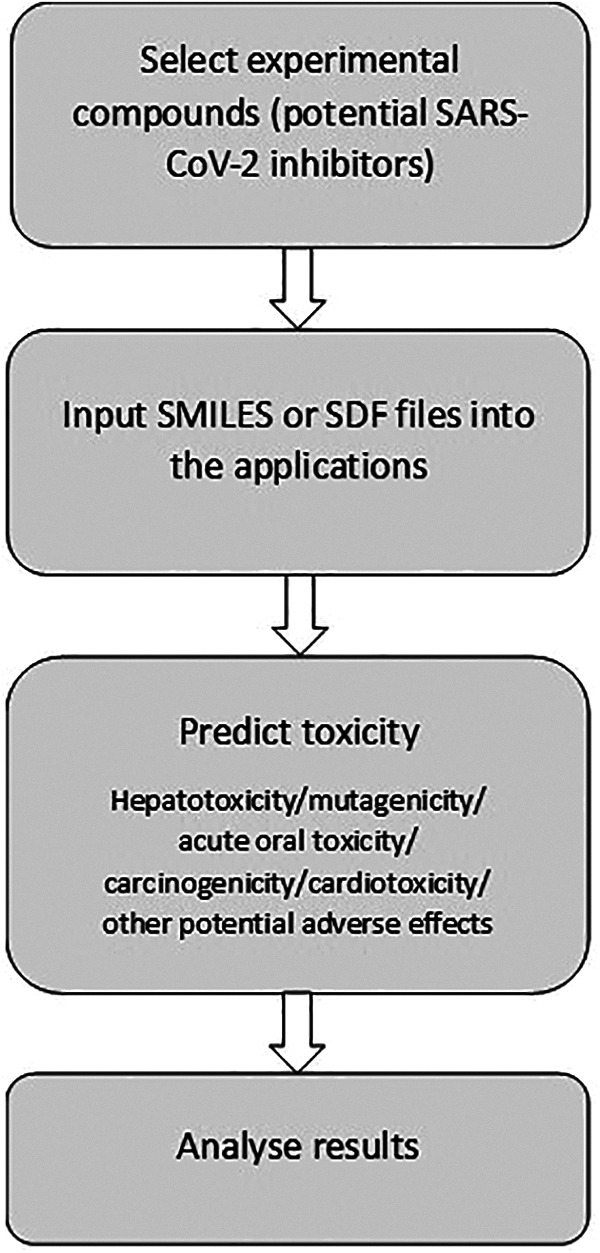
The major steps followed in the prediction of toxicity.

In addition to hepatotoxicity, cardiotoxicity is a commonly observed adverse event that has led to the withdrawal of drugs; one of the causes of drug-induced cardiotoxicity is the blockage of hERG (human Ether-à-go-go-Related Gene) K^+^ channels, resulting in arrhythmia.^42^ In comparison to the number of web-based servers for the prediction of other toxicity endpoints, very few publicly available servers currently exist for cardiotoxicity prediction. Hence, this prediction was performed solely with the Pred-hERG 4.2 server, as it had been trained on a large dataset, displayed the potency of cardiotoxicity, and had shown relatively high accuracy values in earlier studies.^43^ The SMILES representations of the compounds were provided as input to this server; the output displayed the prediction and potency (if found to be positive for cardiotoxicity), along with their respective confidence levels, applicability domain and similar compounds in the dataset. Figure 2 illustrates the main steps followed in the prediction of toxicity. The complete results obtained with all the tools used are shown in *Supplementary Material Table S1*.

## Results and discussion

### Hepatotoxicity

The experimental compounds were categorised according to the results obtained for each parameter. The compounds that were stereoisomers displayed the same results for all the toxicity predictions performed. Most of the compounds were predicted to be possibly hepatotoxic; around 42.1% (8 out of 19) compounds were predicted to be hepatotoxic on all three servers. Only one compound (ZINC000003118440) was negative for hepatotoxicity on two out of the three servers and displayed an unreliable result on VEGA. The compound ZINC000000146942 did not show any result for hepatotoxicity on VEGA. An examination of the hepatotoxicity probability, structural alerts and similar compounds revealed that 11 out of the 19 compounds (57.9%) were similar to a number of sulphonamides that are known to be hepatotoxic. However, the experimental compounds did not possess any sulphur-containing relevant structural alerts. Three out of the 19 compounds (15.8%) were similar to and had a structural alert found in flutamide, a nonsteroidal anti-androgen with a likelihood score of A (i.e. a well-known cause of clinically apparent liver injury) on LiverTox.^44^ Three out of the 19 (15.8%) compounds were similar to, or had the structural alert present in, ximelagatran (Exanta), which is a discontinued anticoagulant.^45^

### Mutagenicity

For the mutagenicity predictions, all the compounds were labelled as ‘non-mutagenic’ from the consensus of results. Only one compound (ZINC001014061061) was predicted to be mutagenic on ProTox-II, while Toxtree showed structural alerts for *S. typhimurium* mutagenicity for two compounds (PubChem CID: 444745 and PubChem CID: 11610052). The structural alerts reported for the compounds were primary aromatic amine, hydroxyl amine and its derived esters, and alkenylbenzene, respectively. However, the results on VEGA varied from low to moderate reliability for almost all of the compounds; the consensus scores reported by the CONSENSUS model ranged from 0.1 to 0.6. Although VEGA reported a result of mutagenicity for 9 out of the 19 compounds (47.4%) by the CONSENSUS model, the consensus scores for both mutagenicity and non-mutagenicity were found to be equal for 7 out of the 9 compounds (77.8% of such cases).

### Acute oral toxicity

For acute oral toxicity, 18 out of the 19 compounds (94.7%) were predicted to have an LD_50_ value above 50 mg/kg on all three servers. The mean prediction accuracy of the results was 63.1% on ProTox-II, and the accuracy ranged from 23% to 69.3%. Notably, only one compound, 444745, was predicted to have an extremely low LD_50_ value of 1 mg/kg on ProTox-II, but the prediction accuracy for this result was only 23%. However, the other two servers, DL-AOT and admetSAR, displayed favourable results, predicting a relatively high LD_50_ value for the same compound.

### Carcinogenicity

Among the compounds considered, 12 out of the 19 compounds (63.2%) were predicted to be carcinogenic on ProTox-II, with probability values ranging from 0.5 to 0.63. Toxtree displayed structural alerts for carcinogenicity for 12 out of the 19 compounds (63.2%); halogenated benzene was the most commonly observed structural alert, appearing in 9 out of the 19 compounds (47.4%). However, it has a positive predictive value of only 31%;^46^ hence, it was not considered a relevant structural alert. On the other hand, the primary aromatic amine, hydroxyl amine, and its derived esters alert, associated with genotoxic carcinogenicity, has a positive predictive power of 81% and was, therefore, labelled a relevant structural alert. Although there was no information on the positive predictive value of the alkenylbenzene and imidazole alerts, numerous alkenylbenzene derivatives are well-known carcinogens. Hence, this was also labelled as a relevant structural alert.^47^ The VEGA results had relatively lower levels of reliability for some compounds — around 68.4% of the compounds showed an applicability domain index value of less than 0.6 for all three models. For 3 out of the 19 compounds (15.8%), the consensus result on VEGA was not equal to the result of the model whose applicability domain index was highest. Around 11 out of the 19 compounds (57.9%) exhibited some degree of similarity to, and/or contained the structural alerts present in, lavoltidine (previously known as loxtidine), which is a histamine 2 receptor antagonist that can induce gastric carcinoid tumours.^48,49^

### Cardiotoxicity and other potential adverse effects

The cardiotoxicity prediction results displayed positive results for potential cardiotoxicity for 3 of the 19 compounds (15.8%), with the potency of the cardiotoxicity being ‘weak or moderate’. Of the 19 compounds, 18 (94.7%) were in the applicability domain of the model; however, the average confidence level was 53.3%. With reference to the toxicity targets, compound 11610052, a 7-hydroxyisoflavone,^50^ showed a high probability of binding to the oestrogen receptor on both admetSAR and VEGA. This compound also exhibited a high probability (0.74) of causing perturbations in mitochondrial membrane potential and a weak probability (0.51, on ProTox-II) of binding to the aryl hydrocarbon receptor. Furthermore, probable aromatase, thyroid receptor, glucocorticoid receptor and androgen receptor binding was predicted by admetSAR, but these results could not be correlated with results from the other tools. Interestingly, previous findings have reported that isoflavones stimulate mitochondrial biogenesis.^51^ This class of compounds is also known to exhibit anti-oestrogenic activity,^52^ which supports the prediction shown here. For some of the compounds, admetSAR predicted a positive result for aromatase, androgen receptor and thyroid receptor binding, but these results were not confirmed with the other tools. Similarly, ZINC000003118440 was predicted on ProTox-II to bind to amine oxidase A and ZINC001014061061 to bind to prostaglandin synthase G/H synthase 1; however, none of the other tools utilised comprised features for binding prediction to these targets, and so a consensus was not obtained.

### Classification

Two tables were created to display the results obtained. Table 3 shows the classification of each compound, based on the in-depth toxicity profiles with all the toxicity endpoints. In this table, each compound is classified into one of three categories according to its predicted toxicity, with Category 1 being ‘least likely to be toxic’ and Category 3 being ‘most likely to be toxic’. The compounds were grouped into these categories based on the criteria shown in Table 2. As all the compounds displayed the same consensus results for acute oral toxicity and mutagenicity, the criteria were defined based on the differences between the other toxicity endpoints (Table 3). Negative results that were associated with the acute oral toxicity and mutagenicity testing, which are part of the safety studies (general toxicity and genotoxicity) recommended by the FDA for pharmaceuticals,^53^ are displayed in Table 4.

**Table 2.** The criteria for classification of the compounds.

Category	Criteria
1	No or low possibility of hepatotoxicity; no or low possibility of carcinogenicity; non-mutagenic; non-cardiotoxic
2	High possibility of hepatotoxicity; high possibility of carcinogenicity; non-mutagenic; possibly cardiotoxic (any one of these criteria)
3	High possibility of hepatotoxicity; high possibility of carcinogenicity; non-mutagenic; possibly cardiotoxic (two or more of these criteria)

**Table 3.** Classification of the 19 compounds, based on their toxicity profiles.

Category	Compound	Key results^a^
1	ZINC000152979101	Similar to sulphaphenazole, celecoxib and lavoltidine; no relevant structural alerts
1	ZINC001627499877	Similar to sulphaphenazole and celecoxib; no relevant structural alerts
1	ZINC000544491494	Similar to celecoxib, gliclazide, rosiglitazone, lavoltidine, granisetron and pirinixil; no relevant structural alerts
1	ZINC000544491491	Similar to celecoxib, granisetron, sulphaphenazole, lavoltidine, granisetron and pirinixil; no relevant structural alerts
1	ZINC000543523838	Similar to sulphaphenazole, rosiglitazone, celecoxib, lavoltidine and granisetron; no relevant structural alerts
1	ZINC000543523837	Similar to sulphaphenazole, rosiglitazone, celecoxib, lavoltidine and granisetron; no relevant structural alerts
1	PubChem CID: 444743	Similar to ximelagatran; no relevant structural alerts
1	ZINC001014061061	Similar to perindopril, enalapril and ximelagatran; no relevant structural alerts
1	ZINC001362111980	Similar to sulphaphenazole, celecoxib and pirinixil; no relevant structural alerts
1	ZINC000003118440	Similar to dacarbazine; has relevant structural alert (dacarbazine)
2	ZINC000541676760	Similar to celecoxib; has relevant structural alert (flutamide, similarity = 0.742)
2	ZINC000541677852	Similar to celecoxib and lavoltidine; has relevant structural alert (flutamide, similarity = 0.732)
2	ZINC000152975931	Similar to celecoxib (0.802) and flutamide; no relevant structural alerts
2	ZINC001014061081	Similar to lavoltidine and granisetron; has relevant structural alert (lavoltidine)
2	PubChem CID: 11610052	Similar to dicumarol, acenocoumarol, flavopiridol, cianidanol, sterigmatocystin and quercetin; has relevant structural alert; binding to oestrogen receptor
3	PubChem CID: 444603	Similar to amprenavir and ximelagatran; has relevant structural alert (ximelagatran); possibly cardiotoxic
3	ZINC000636416501	Similar to oxyphenisatin acetate, phenisatin, celecoxib, ezetimibe and aripiprazole; has relevant structural alerts; possibly cardiotoxic
3	PubChem CID: 444745	Similar to lopinavir; has relevant structural alert; was positive for carcinogenicity on all the three servers; possibly cardiotoxic
—	ZINC000000146942	Similar to phenobarbital and primidone; has relevant structural alerts; insufficient information to determine hepatotoxicity, so category prediction was not made

^a^ Similar compounds listed in the ‘Key results’ column have > 0.75 similarity to the experimental compounds.

Category 1 means ‘least likely to be toxic’, while Category 3 means ‘most likely to be toxic’.

**Table 4.** The negative results for acute oral toxicity and mutagenicity.

Compound	Result	Server/application	Reliability
ZINC000636416501	Mutagenic	VEGA	Moderate
ZINC001362111980	Mutagenic	VEGA	Low
PubChem CID: 444745	Fatal if swallowed; LD_50_ 1 mg/kg	ProTox-II	Low
	Structural alert for mutagenicity (primary aromatic amine, hydroxyl amine and its derived esters)	Toxtree	N/A
ZINC001014061061	Mutagenic	ProTox-II	N/A; probability = 0.52
PubChem CID: 11610052	Structural alert for mutagenicity (alkenylbenzene)	Toxtree	N/A

N/A = not applicable.

In Table 3, Category 1 comprises 10 compounds (10/19; 52.6%). The compounds in this category exhibited relatively high (0.75–0.8) similarity to compounds/drugs such as sulphaphenazole, celecoxib, lavoltidine and granisetron, but did not have any relevant structural alerts that are present in these compounds/drugs. In addition, these compounds were non-mutagenic and non-cardiotoxic. Overall, the toxicity profiles of all the compounds in this category were largely promising.

Category 2 consists of five compounds (5/19; 26.3%), which were ranked primarily due to their high degree of similarity to, or the presence of structural alerts commonly found in, flutamide, celecoxib (likelihood score of B on LiverTox^54^), ximelagatran and lavoltidine. Compound 11610052 was also placed in this category due to its potential binding to the oestrogen receptor; such binding to nuclear hormone receptors can elicit serious off-target effects in other physiological processes.^55^

Lastly, three compounds (3/19; 15.8%) were classified under Category 3; all three such compounds had been labelled as possibly cardiotoxic. ZINC000636416501 displayed some of the structural alerts found in oxyphenisatin acetate and lumiracoxib, which were previously withdrawn from the market due to cases of hepatotoxicity.^56,57^ Compound 444603 exhibited similarity with and had the structural alert present in ximelagatran. Similarly, compound 444745 had more than four structural alerts for carcinogenicity on VEGA and Toxtree combined, and a moderate probability of 0.58 for carcinogenicity on ProTox-II.

Lastly, compound ZINC000000146942 was not classified due to insufficient data to determine the hepatotoxicity prediction; while DL-DILI predicted positively for hepatotoxicity, ProTox-II indicated a negative result, and there was no result on VEGA (note that VEGA does not display any results, if the compound has none of the structural alerts used in the prediction model).

As the general consensus of results indicated mostly negative results for acute oral toxicity and mutagenicity testing, Table 4 lists the red flags identified with respect to these toxicity endpoints, along with the server/application utilised and the level of reliability, if available. Those compounds not listed in Table 4 showed negative results for these toxicity endpoints.

### Limitations of the study

Although we were able to gain a detailed toxicity profile for the 19 compounds, based on multiple significant toxicity endpoints, our study has a few limitations. Since we utilised only one tool for the prediction of hERG inhibition, these results may not be as reliable as those obtained with three tools and by applying the consensus approach. Moreover, due to the unavailability of web-based servers/applications or unreliability of results, we were unable to appropriately evaluate reproductive toxicity, which is also recommended by regulatory agencies such as the FDA.^53^ Further *in vitro* and *in vivo* studies with respect to these endpoints will help to obtain the complete toxicity profiles of these compounds, and further the understanding of any adverse effects that they may exhibit.

## Conclusions

While new drug development requires more time and investment in contrast to existing drug repurposing,^58^ the results obtained through the *in silico* prediction of toxicity can accelerate the process of development, reduce costs,^59^ and pave the way for drugs that exhibit favourable safety and efficacy to combat Covid-19. Overall, the results of the current study indicate that a number of potentially useful experimental compounds have promising toxicity profiles, when compared to existing drugs currently being repurposed for Covid-19 treatment, including remdesivir, chloroquine and favipiravir.^9^

In this study, *in silico* tools were used to obtain toxicity predictions for a number of experimental compounds that can potentially bind to proteins in SARS-CoV-2. A range of web-based servers were used to evaluate different toxicity endpoints, obtain consensus results, and categorise the compounds according to a set of defined criteria. A majority of the compounds exhibited encouraging toxicity profiles with respect to the studied endpoints; 10 out of the 19 compounds were associated with low probabilities of hepatotoxicity and carcinogenicity, and were predicted to be non-mutagenic and non-cardiotoxic. As efforts are ramped up to find SARS-CoV-2-specific therapies, these results can hopefully assist lead optimisation by providing an indication of how the compounds may fare during *in vitro* and *in vivo* toxicity testing, and their potential to be safe and effective drugs against Covid-19.

## Supplementary material

Supplemental Material, sj-xlsx-1-atl-10.1177_02611929211008196 - The Use of *In Silico* Tools for the Toxicity Prediction of Potential Inhibitors of SARS-CoV-2 Click here for additional data file. Supplemental Material, sj-xlsx-1-atl-10.1177_02611929211008196 for The Use of *In Silico* Tools for the Toxicity Prediction of Potential Inhibitors of SARS-CoV-2 by Varsha Bhat and Jhinuk Chatterjee in Alternatives to Laboratory Animals
